# Isolation of *OsMetAP10*, a Peptidase_M24 Superfamily Gene, Regulating Heading Date in Rice

**DOI:** 10.3390/biology14020178

**Published:** 2025-02-10

**Authors:** Quanyi Sun, Jianhua Zhao, Guangda Wang, Yu Wang, Yuntao Zhu, Yu Yan, Zihang Chen, Zongxiang Chen, Zhiming Feng, Shimin Zuo

**Affiliations:** 1 Jiangsu Key Laboratory of Crop Genomics and Molecular Breeding/Zhongshan Biological Breeding Laboratory/Key Laboratory of Plant Functional Genomics of the Ministry of Education, Agricultural College of Yangzhou University, Yangzhou 225009, China; sunquanyi2023@163.com (Q.S.); dx120180075@stu.yzu.edu.cn (J.Z.); dx120210125@stu.yzu.edu.cn (G.W.); zyt2904734346@163.com (Y.Z.); y.0307@outlook.com (Y.Y.); 19825864560@163.com (Z.C.); czx@yzu.edu.cn (Z.C.); fengzm@yzu.edu.cn (Z.F.); 2 Biotechnology Research Institute, Shanghai Academy of Agricultural Sciences, Shanghai 201106, China; 3 Jiangsu Co-Innovation Center for Modern Production Technology of Grain Crops/Jiangsu Key Laboratory of Crop Genetics and Physiology, Yangzhou University, Yangzhou 225009, China; 4 Joint International Research Laboratory of Agriculture and Agri-Product Safety, Ministry of Education of China/Institutes of Agricultural Science and Technology Development, Yangzhou University, Yangzhou 225009, China

**Keywords:** rice (*Oryza sativa* L.), late-heading-date mutant, gene isolation, *OsMetAP10*, peptidase_M24 superfamily

## Abstract

The heading date is one of the important traits in rice, which greatly affects grain yield and regional adaptability. Although the flowering pathways in rice have been extensively investigated, the genes involved in flowering remain largely unknown. Here, we report a rice *lhd* mutant, which showed late flowering under both long-day (LD) and short-day (SD) conditions. Through MutMap+ and linkage analysis, a deletion mutation in *OsMetAP10* was inferred as the most likely candidate for *lhd* late flowering. *OsMetAP10* encodes a methionine aminopeptidase that belongs to the peptidase_M24 subfamily III. The *OsMetAP10* gene is constitutively expressed in rice and is induced by light, with a rhythmic expression pattern. *OsMetAP10* knockout lines displayed late heading as the *lhd* mutation, while no alternations in morphology and heading were observed on *OsMetAP10* overexpression lines, further confirming the mutation of *OsMetAP10* as responsible for the late heading of *lhd*. Through RT-qPCR and transcriptome analysis, we revealed that the upregulated expression of the *FT-like* gene *OsFLT4*, a negatively flowering regulator, and the downregulation of flower development-related genes, *OsMADS14*, *OsMADS15*, and *OsMADS34*, played critical roles in determining the late flowering of the *OsMetAP10* mutation. This study reports a new gene affecting flowering and provides a new insight into the role of *OsMetAP10* in regulating rice heading.

## Simple Summary

The peptidase_M24 protein family is highly conserved in eukaryotes and has been reported to play important roles in plant development and response to abiotic stress in *Arabidopsis*. However, few studies have reported its function in rice to date. In this article, we identified a rice mutant, *lhd*, with a delayed heading phenotype, and then through the MutMap+-based gene-mapping approach and sequence analysis, we found that a peptidase_M24 protein-encoding gene, *OsMetAP10*, was associated with the late-heading phenotype of the *lhd* mutant. By using CRISPR-Cas9 technology and transgenic overexpression, we obtained three *OsMetAP10* knockout lines and three *OsMetAP10* overexpression lines, and we confirmed that *OsMetAP10* is involved in regulation of rice flowering. Through RT-qPCR and transcriptome analysis, we inferred the downstream signaling of *OsMetAP10* mutation on later flowering. The OsMetAP10 protein was localized in both the cytoplasm and nucleus and constitutively expressed in different development stages and various tissues in rice. This study provides insight into the role of *OsMetAP10* in the regulation of rice heading.

## 1. Introduction

Rice (*Oryza sativa* L.) is one of the most important crops in the world, and its yield directly affects half of the world’s population. The heading date (or flowering time) is an important agronomic trait of rice that is closely related to the yield [1]. Rice is a short-day (SD) plant that blooms under photoperiodic influence after passing the basic vegetative growth phase (BVP) [2], flowering earlier under SD conditions and later under long-day (LD) conditions. In addition, the heading period of rice is also affected by external factors, such as temperature and nutrient supply [3]. In contrast to *Arabidopsis thaliana*, which has only one florigen gene, *FLOWERING LOCUS T* (*FT*), two florigen genes have been reported in rice, named *Heading date 3a* (*Hd3a*) and *RICE FLOWERING LOCUS T1* (*RFT1*) [4]. *Hd3a* and *RFT1* both encode a phosphatidylethanolamine binding protein homologous to the *Arabidopsis FT* protein, which is delivered to the shoot apical meristem (SAM) through the phloem after leaf induction [4,5]. In the cytoplasm of the SAM, Hd3a interacts with the *14-3-3* protein and translocates to the nucleus, forming a flowering activation complex (FAC) with the bzip transcription factor *OsFD1* [6,7]. The FAC stimulates the expression of the flower development-related genes *OsMADS14*, *OsMADS15*, *OsMADS18*, and *OsMADS34* to initiate the floral transition in rice [8,9]. The related molecular mechanism of florigen under LD conditions is still incomplete. Recently, it has been reported that phosphorylation of OsFD1 by OsCIPK3 can promote the formation of a RFT1-containing FAC to induce flowering under LDs in rice [10].

Two photoperiodic flowering pathways have been reported in rice. One is the *OsGIGANTEA* (*OsGI*)–*Heading date 1* (*Hd1*)–*Hd3a*/*RFT1* pathway, which is similar to the *GIGANTEA* (*GI*)–*CONSTANS* (*CO*)–*FT* pathway in *Arabidopsis Thaliana* [11]. *OsGI* is a homologue of Arabidopsis *GI* (*At1g22770*), which is associated with the circadian clock in rice. *OsGI* can promote the expression of *Hd1* and flowering in SDs [12]. *Hd1* is a homologue of the Arabidopsis flowering integration factor *CO*, the rhythm of which is regulated by the upstream *OsGI* [12]. *Hd1* inhibits *Hd3a* expression under LDs, but promotes flowering by activating *Hd3a* expression under SD conditions [13]. In addition, *Hd1* mediates the transcriptional regulation of *Hd3a* by interacting with Days to heading on chromosome 8 (DTH8) to form the DTH8-HD1 module under different light lengths [14]. The other unique photoperiodic flowering pathway in rice is *Grain number, plant height, and heading date 7* (*Ghd7*)*–Early heading date 1* (*Ehd1*)*–Hd3a/RFT1* [4]. *Ghd7* is a transcriptional repressor whose expression increases with the day length [15]. Overexpression of *Ghd7* delays flowering in rice under LDs [16]. *Ehd1* encodes a *B*-type response regulator, which is the core regulator of *Hd3a/RFT1*, and can promote *Hd3a/RFT1* expression in both SDs and LDs [17]. *Ehd1* integrates the upstream flowering signal of rice, which is positively regulated by *OsGI*, *Ehd2*, and *Ehd4* genes under SDs and LDs [18]. *OsCONSTANS-LIKE 4* (*OsCOL4*), which is independent of photoperiod regulation, inhibits flowering by suppressing the expression of *Ehd1* [19]. Furthermore, *Hd1* can promote the expression of *Ghd7* and be recruited by *Ghd7* to form a complex, which suppresses the expression pathway of *Ehd1–Hd3a/RFT1* by binding to the cis-regulatory sequence of the *Ehd1* promoter in LDs [20]. The two flowering pathways explain the molecular mechanism of rice heading changes to some extent. However, due to the complexity of the rice heading control mechanism, there are still many related mechanisms and molecular mechanisms that have not been studied.

The peptidase_M24 protein superfamily is highly conserved in eukaryotes and consists of many different types of proteins, which usually have a ‘pita bread’ fold structure containing both alpha helices and an anti-parallel beta sheet [21]. They are mainly involved in protein synthesis and metabolism, maintenance of chloroplast structure and function, and signal transmission [22]. Recently, peptidase_M24 protein family members have been reported to play a role in plant growth and development and abiotic stress response. In eukaryotes, the majority of mature proteins undergo N-terminal methionine (Met) excision (NME) [23]. Six methionine aminopeptidase (MAP) proteins have been discovered in *Arabidopsis thaliana*, among which three have been confirmed as components of the NME mechanism [23]. *Arabidopsis EBP1* (*AtEBP1*) is phosphorylated by kinase and binds to the promoter region of *calmodulin-like protein 38* (*CLM38*), which is involved in signaling in Arabidopsis [24]. *HvMETHIONINE AMINOPEPTIDASE* (*HvMAP*) plays a role in freeze tolerance by promoting protein maturation [25]. However, the systematic identification and functional reports of peptidase_M24 protein family members have not been discovered in rice, thus further research is required.

In this study, we identified a rice heading date gene named *OsMetAP10*. The mutant form of this gene causes flowering approximately 10 days later than wild-type (WT) plants under both SDs and LDs. Similar to many genes that have been reported to control the heading date of rice in the past, mutations in this gene will affect the traits related to rice yield. *OsMetAP10* encodes a methionine aminopeptidase that contains zf-C6H2 and peptidase_M24 domains, which is located in the cytoplasm and nucleus, belonging to the peptidase_M24 superfamily. Our study demonstrated that the *OsMetAP10* gene was expressed constitutively and rhythmically in rice. Further studies showed that the *OsMetAP10* mutation can increase the expression of *OsFTL4* and decrease the expression of *OsMADS14*, *OsMADS15*, and *OsMADS34* in the SAM of rice. These results indicate that the *OsMetAP10* gene is a new heading date regulator and influences agronomic traits. Our study enriches the genetic regulatory network of flowering-time genes and provides a theoretical basis and genetic resources for rice adaptive breeding.

## 2. Materials and Methods

### 2.1. Plant Materials and Growth Conditions

The *lhd* mutant was sourced from an ethyl methane sulfonate (EMS)-induced mutant library of the japonica cultivar Dongjin (*Oryza sativa* L. *subsp. japonica*). Rice plants were cultivated in the experimental paddy fields under natural long-day (NLD) conditions at Yangzhou University (119°24′ E, 32°23′ N) and natural short-day (NSD) conditions in Lingshui, Hainan, China (110°1′ E, 18°30′ N), in different growing seasons. The average length of sunshine in the experimental field of Yangzhou University exceeded 13 h from June to August, while the average length of sunshine in Lingshui, Hainan, was less than 12 h from December to February (the data of day length were collected from www.timeanddate.com, accessed on 18 November 2024.).

### 2.2. Population Construction and Genome Sequencing

We crossed the *lhd* mutant with its progenitor wild-type (WT) line Dongjin, and the resulting F_1_ progeny was further self-pollinated to generate a F_2_ population. Individual plants with normal heading dates were selected from the aforementioned F_2_ population and self-pollinated to obtain multiple F_3_ populations. Among them, the population with a 3:1 phenotypic segregation ratio of heading dates was chosen. By evaluating the heading date of each individual’s progeny within the selected F_3_ populations, they were classified into a normal-heading phenotype pool (similar to the WT) and a late-heading phenotype pool (similar to *lhd*). Fresh leaves from thirty randomly selected individuals were sampled from each phenotype pool for DNA extraction and then mixed in equal proportions. In this study, the EDTA rapid extraction method was employed for DNA extraction. For the next-generation resequencing, paired-end (PE) sequencing was carried out on the Illumina sequencing platform with a 30× sequencing depth (BENAGEN, Wuhan, China). The raw data were quality-controlled for reads using fastp (version 0.12.6). With the Nipponbare genome (https://ftp.ensemblgenomes.ebi.ac.uk/pub/plants/release-60/fasta/oryza_sativa, accessed on 28 September 2023.) as the reference genome, BWA (v0.7.17) and Samtools software (v1.14) in Linux were used to build an index and align the reads. SNPs/InDels were called by GATK software (v4.2.6.0), and the heterozygous loci and variant loci with a read number less than 20 were filtered out.

### 2.3. MutMap+ Analysis and Phenotype Association Verification

We calculated the ΔSNP index and ΔInDel index as previously described in the MutMap+ method [26]. A sliding window with a window size of 0.5 Mb and step size of 10 Kb was set, and R language (4.4.1) was used to calculate the average ΔSNP and ΔInDel indices located in the window. We set the threshold as the mean (all ΔSNP index or ΔInDel index) ± three times the standard deviation (all ΔSNP index or ΔInDel index), as previously described [27]. To verify the association between the candidate mutation in *Os10g0508400* and the late-heading phenotype of the *lhd* mutant, WT and late-heading phenotype plants from the F3 population isolated by the heading stage phenotype were further genotyped using direct sequencing of the PCR products amplified by the primer set 8400-F/R (Table S1).

### 2.4. Plasmid Construction and Transformation

With the CRISPR-P v2.0 (http://crispr.hzau.edu.cn/CRISPR2/, accessed on 4 April 2022) website, we set gene targets, putting it in vector pOs-sgRNA. We performed the Gateway cloning LR reaction (Invitrogen, Carlsbad, CA, USA) on the obtained pOs-sgRNA construct using the pH-Ubi-cas9-7 vector to generate sgRNA:Cas9, targeting *OsMetAP10*, as previously described [28]. The recombinant plasmid was introduced into Agrobacterium EHA105 and then transformed into Dongjin callus. Primer 8400-F/R was used to detect the T0 generation. Primers used in this assay are listed in Table S1.

To construct an overexpression vector of *OsMetAP10*, the ClonEapress Ultra One Step Cloning Kit V3 (Vazyme, No. C117-02) was used to clone *OsMetAP10*-CDS into pCubi1390, an overexpression vector digested by Pst1. The overexpression vector pCubi1390 contains an enhanced CaMV 35S promoter, and the end of the insertion site is tagged with 3 × Flag (Figure S1). The recombinant plasmid was introduced into Agrobacterium strain EHA105 and then transformed into Dongjin callus. Relevant primers are listed in Table S1.

### 2.5. Phylogenetic Analysis

The reference genomes and protein sequences of monocotyledonous plants (rice, sorghum, wheat, and maize) and dicotyledonous plants (*Arabidopsis thaliana*, cucumber, *Gossypium raimondii*, and soybean) were downloaded from the Rice Annotation Project (RAP) (https://rapdb.dna.affrc.go.jp/, accessed on 12 October 2024.). Identification of *OsMetAP10* homologous genes in rice and other varieties was performed using the Quick Find Best Homology plug-in with default parameters in TBtools II [29]. MEGA11 software and Trimal were used to compare and trim the protein sequences. The existing hidden Markov model (HMM) of the *OsMetAP10* gene family (Pfam: PF0057, https://www.ebi.ac.uk/interpro/, accessed on 16 October 2024.) was employed to identify the members of the gene family in rice and Arabidopsis. The phylogenetic trees were generated using the neighbor-joining method with the default parameters, with the exception of 1000 bootstrap replications in MEGA11 software. MEME (https://meme-suite.org/meme/, accessed on 16 October 2024.) and NCBI CD-Search (https://www.ncbi.nlm.nih.gov/Structure/bwrpsb/bwrpsb.cgi, accessed on 16 October 2024) tools were used to analyze the motifs and domains of this gene family, respectively. Images such as the phylogenetic trees were edited using the Interactive Tree of Life online tool (http://itol.embl.de/, accessed on 16 October 2024.) and R (version 4.4.1).

### 2.6. Histochemical GUS (β-Glucuronidase) Staining

To construct promoter-GUS transgenic lines, the pOsMetAP10:GUS plasmid was generated by amplifying the promoter of OsMetAP10 using primers PMetAP10-F/R (Table S1). Then, the vectors were transferred into WT plants. According to the manufacturer’s manual, GUS staining solution was configured with the GUS staining substrate x-glux (Solarbio, Beijing, China, ID:x8060) to detect GUS activity.

Different tissues of the Pro-*OsMetAP10*:GUS transgenic plants at different stages (seedling, tillering, booting, and heading) were placed in β-GUS staining solution overnight at 37 °C, and then eluted in 75% ethanol several times. We checked them and took pictures at the end.

### 2.7. Diurnal Expression Analysis

WT seeds were sown in long-strip pots and cultivated under SD (10 h light, 28 °C/14 h dark, 26 °C; relative humidity of 65%) or LD (14 h light, 28 °C/10 h dark, 26 °C; relative humidity of 65%) conditions for 30 days. Leaves were harvested every 4 h starting from 0:00 am for 24 h. For each time point, the topmost expanded leaf from three different individuals was collected as a biological replicate.

### 2.8. Total RNA Extraction, Reverse Transcription, and Quantitative Real-Time PCR

Total RNA was isolated using FreeZol Reagent (Vazyme, Nanjing China; ID:R711-01) according to the manufacturer’s instructions. RNA reverse transcription was carried out by employing the HiScript III RT SuperMix for qPCR (Vazyme, China; ID:R323-01) for the synthesis of the first-strand cDNA. The acquired expression value data were subjected to transformation and analysis by means of the 2^−ΔΔct^ method. The reference gene, *Ubiquitin* (*LOC_Os05g06770*), was chosen, and its forward and reverse primer sequences for RT-qPCR were F: CCTGGCTGACTACAACATC and R: AGTTGACAGCCCTAGGGTG, respectively. Three biological replicates and three technical replicates were set for the samples.

### 2.9. Transient Expression Assay in Rice Protoplasts

First, we amplified the full-length coding sequence of *OsMetAP10* and cloned it into PAN580-GFP. We put 5–10 μg each of PAN580-GFP and OsMetAP10-GFP plasmids into a 2 mL centrifuge tube. Then, we added 100 μL of the ready-made protoplast solution and shook well. Next, we added 110 μL of PEG solution and flicked gently to mix. It was then incubated in the dark at room temperature for 10 min. Then, we added 440 μL of W5 solution and mixed. After centrifuging at 200 g for 3 min, we aspirated the supernatant carefully. Finally, we resuspended it in 300 μL of WI solution (0.5 M mannitol, 20 mM KCl, 4 mM MES, pH 5.7) and incubated at room temperature under light or dark for 6–16 h. We used the LSM880 laser confocal microscope at Yangzhou University Testing Center for observation and imaging.

### 2.10. RNA-Seq Analysis

RNA was extracted from the shoot apical meristems of the WT and *lhd*, with three biological replicates set up. It was sequenced on Illumina (BENAGEN, Wuhan, China) with a 30× depth. We used fastp (Linux) for QC, Hisat2 and Samtools for alignment, and featureCounts for counting. We standardized with DESeq2 (R) and screened DEGs using “log_2_Foldchange > 0.5, *p*-adjust < 0.05”.

## 3. Results

### 3.1. Identification of a Rice Late-Heading-Date Mutant

**Figure 1:**
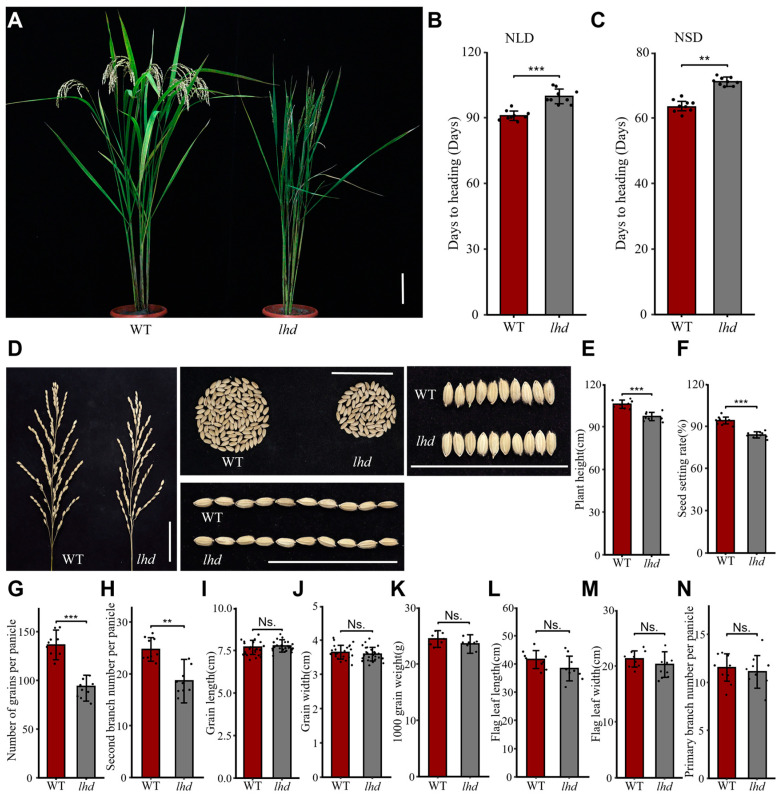
Phenotypes of wild-type (WT) and the *lhd* mutant. (**A**) Plant phenotypes of WT and the *lhd* mutant under NLD conditions. Scale bar = 10 cm. (**B**,**C**) Days to heading of WT and the *lhd* mutant under NLD (**B**) and NSD (**C**) conditions. (**D**) Grain phenotypes of WT and *lhd*. Scale bar = 5 cm. (**E**–**N**) Comparison of agronomically important traits between WT and *lhd*. ** and *** indicate statistically significant differences at *p* < 0.01 and *p* < 0.001 (Student’s *t* test), respectively, and Ns. indicates no statistically significant difference.

In an EMS-induced Dongjin (DJ) rice mutant library, we found a late-heading-date mutant, named *lhd* (Figure 1A), which showed that the days to heading was 99.8 ± 3.3 days, apparently later than the DJ wild-type (WT) of 90.9 ± 2.2 days under the natural long-day (NLD) condition in Yangzhou (Figure 1B). To investigate the heading date of *lhd* under the natural short-day (NSD) condition, we then planted *lhd* and WT in Lingshui, Hainan Province, and found that the *lhd* mutant (63.3 ± 3.1 days) was 8.3 days later than that of WT (71.6 ± 1.6 days; Figure 1C). Besides the heading date, we also found that several agronomical traits, like plant height, seed setting rate, grain number per panicle, and the second branch number per panicle, were significantly reduced in the *lhd* mutant compared to WT (Figure 1E–H and Table S2). However, no significant differences were found in grain length, grain width, 1000-grain weight, flag leaf length, flag leaf width, and the primary branch number per panicle between *lhd* and WT (Figure 1I–N and Table S2). Together, these data indicate that the *lhd* mutant delayed the flowering time under both NSD and NLD conditions, as well as decreased several agronomical traits.

### 3.2. Gene Mapping and Candidate Gene Analysis for Late Flowering of lhd Mutant

To identify the gene responsible for the delayed flowering in the *lhd* mutant, we crossed the *lhd* with the WT and observed that all F_1_ plants showed normal heading for the WT under the NLD condition in Yangzhou. Then, the F_1_ was self-crossed to generate the F_2_ population, and statistical analysis was conducted on the heading date of 200 F_2_ plants under the NLD condition in Yangzhou. It was observed that the segregation of normal heading and delayed heading plants followed a 3:1 ratio (156:44, χ^2^ = 0.96, P_df = 2_ = 0.6188 > 0.05). Together, these data indicated that the late-heading phenotype of *lhd* is controlled by a monogenic recessive gene. To map the gene for late heading, we then harvested as many F_3_ seeds as possible from individual F_2_ plants with the normal heading date as the WT plant. The F_3_ families showing segregation for WT and mutant phenotypes were selected, in which the total genomic DNA of 30 F_3_ individuals with the WT phenotype and 30 F_3_ individuals with the mutant phenotype were extracted, respectively, to make two bulks of DNA for next-generation sequencing.

**Figure 2:**
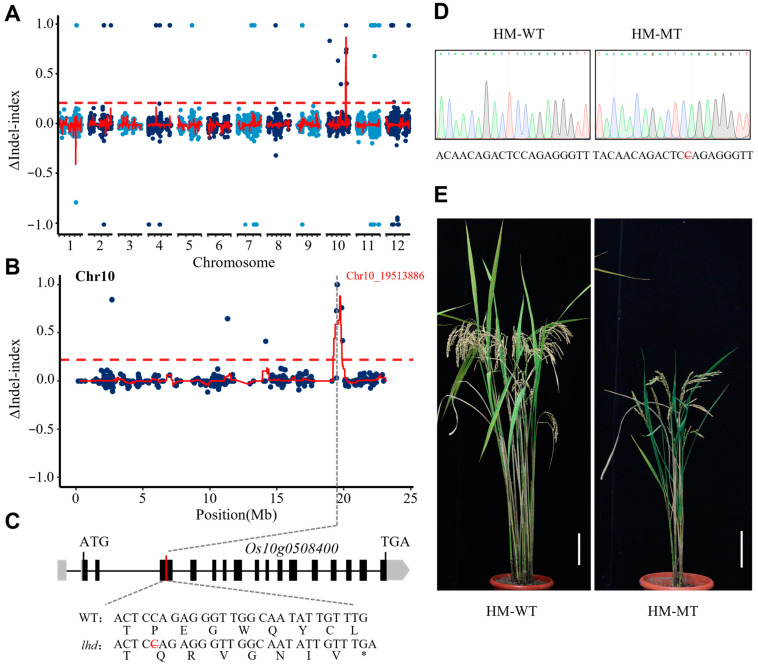
Linkage analysis and candidate gene analysis for the late-heading phenotype in the *lhd* mutant. (**A**) Distribution map of the ΔInDel index on 12 rice chromosomes. (**B**) Distribution map of the ΔInDel index on chromosome 10. The red broken lines in (**A**,**B**) represent the average value of the ΔInDel index in the window, calculated by sliding window. The threshold (0.22) is represented by a reddish-brown dotted line and is obtained by calculating the average value of all ΔInDel indices plus three times the standard deviation of all ΔInDel indices. (**C**) Gene structure of *Os10g0508400*. The red line indicates the position of the InDel Chr10_19513884 in the gene that caused an amino acid change, leading to premature termination of the protein. UTR and CDS are filled with gray and black, respectively. * represents a stop codon. (**D**) Original sequencing map confirming the mutation site in the *Os10g0508400* gene using the plants from the *lhd*/WT F_3_ segregation family population. HM-WT and HM-MT represent the homozygous wild-type genotype and the homozygous mutant genotype, respectively. (**E**) Comparison of HM-WT and HM-MT plants at the maturity stage. Scale bar = 10 cm.

Based on the resequencing data and MutMap+ method, we identified a single unique region between 18.51 and 20.33 Mb on chromosome 10 showing a peak of the InDel index close to 1 in the mutant bulk, but it was missing in the wild-type bulk (Figure 2A and Figure S2). Furthermore, a total of 4 InDels (insertions and deletions) with an ΔInDel index > 0.22 within the region for the mutant bulk were identified (Figure 2B and Table S2). Among them, only one InDel, Chr10_19513886, located at 19513886 bp on chromosome 10, was a nonsynonymous variation, leading to an amino acid change (Table S2) in the *Os10g0508400* gene, which resulted in its protein’s premature termination (Figure 2C). Subsequently, we further sequenced 200 more F_3_ plants with the late-heading phenotype from the F_3_ segregation families, and we confirmed that Chr10_19513886 was completely co-segregated with the late-heading phenotype, indicating that the mutation of *Os10g5058400* is the most likely candidate gene for the *lhd* late-heading phenotype (Figure 2D,E).

### 3.3. Phylogenetic Analysis for Os10g0508400 Homologous Genes

**Figure 3:**
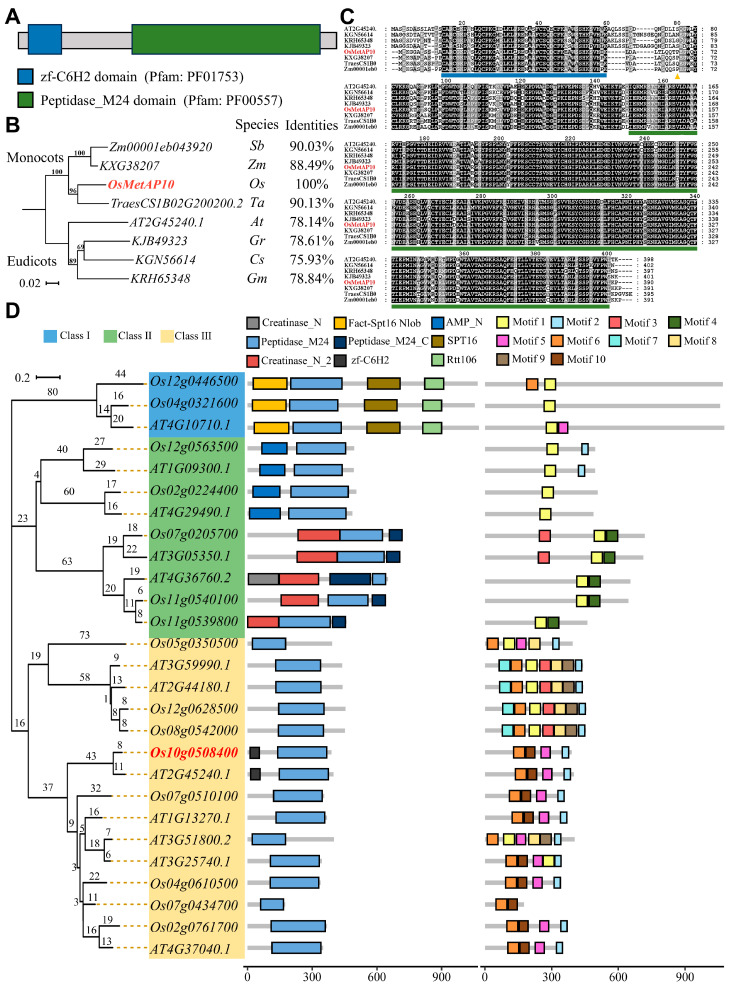
Protein structure of OsMetAP10 and its phylogenetic analysis with homologous proteins. (**A**) Schematic diagram of the functional domains in OsMetAP10 protein. The domains were detected using the Simple Modular Architecture Research Tool (http://smart.embl-heidelberg.de/, accessed on 16 October 2024). (**B**) Phylogenetic tree constructed with OsMetAP10 and its homologous proteins from different species. Eudicotyledon plants: *At*, *Arabidopsis thaliana*; *Cs*, *Cucumis sativus*; *Gr*, *Gossypium raimondii*; *Gm*, *Glycine max*. Monocotyledonous plants: *Sb*, *Sorghum bicolor*; *Zm*, *Zea mays*; *Os*, *Oryza sativa* L.; *Ta*, *Triticum aestivum*. The numbers at the nodes indicate the bootstrap values of the branches. The eigenvalues represent the sequence similarity between the OsMetAP10 protein and its homologous proteins, and the protein sequence alignment was performed using The Sequence Manipulation Suite [31]. (**C**) Multiple sequence alignment for OsMetAP10 homologous proteins. The blue and green lines indicate the zf-C6H2 domain and peptidase_M24 domain, respectively. The orange triangle indicates the amino acid site where the OsMetAP10 protein was mutated in the *lhd* mutant. (**D**) Evolutionary tree, domain, and motif analysis of the peptidase_M24 gene family in *Arabidopsis thaliana* and rice. Fifteen peptidase_M24 protein members in rice and twelve in *Arabidopsis thaliana* were compared, and the tree was constructed using the neighbor-joining method (1000 replicates) in Mega11 software. *OsMetAP10/Os110g0508400 is* highlighted in red font. The numbers at the nodes indicate the bootstrap values of the branches.

The *Os10g0508400* gene was annotated to encode a methionine aminopeptidase. According to the previous naming rules and its location on the chromosome, this gene was named *OsMetAP10* [30]. Protein sequence analysis showed that OsMetAP10 encompasses a zf-C6H2 domain at its N-terminal and a peptidase_M24 domain at its C-terminal (Figure 3A). The phylogenetic analysis showed that the OsMetAP10 protein has a high degree of similarity to its homologous proteins in both dicotyledonous and monocotyledonous plants, indicating the high conservation of this protein (Figure 3B,C). The *OsMetAP10* gene belongs to the peptidase_M24 superfamily, though few studies previously investigated its gene family and function in rice [22]. By using the peptidase_M24 sequence, we identified 15 putative members containing the peptidase_M24 domain in rice and constructed a neighbor-joining (NJ) phylogenetic tree with 12 peptidase_M24-containing proteins from Arabidopsis (Figure 3D and Figure S3). Based on the combined analysis of motifs and domains, the peptidase_M24 proteins were classified into three subfamilies. The protein sequences of subfamily ΙΙΙ, harboring OsMetAP10, all contained motif 2 and motif 6, indicating that OsMetAP10 belongs to peptidase_M24 protein subfamily III (Figure 3D and Table S3).

### 3.4. OsMetAP10 Mutation Accounts for the Late Heading and Inferior Performance of lhd Mutant

**Figure 4:**
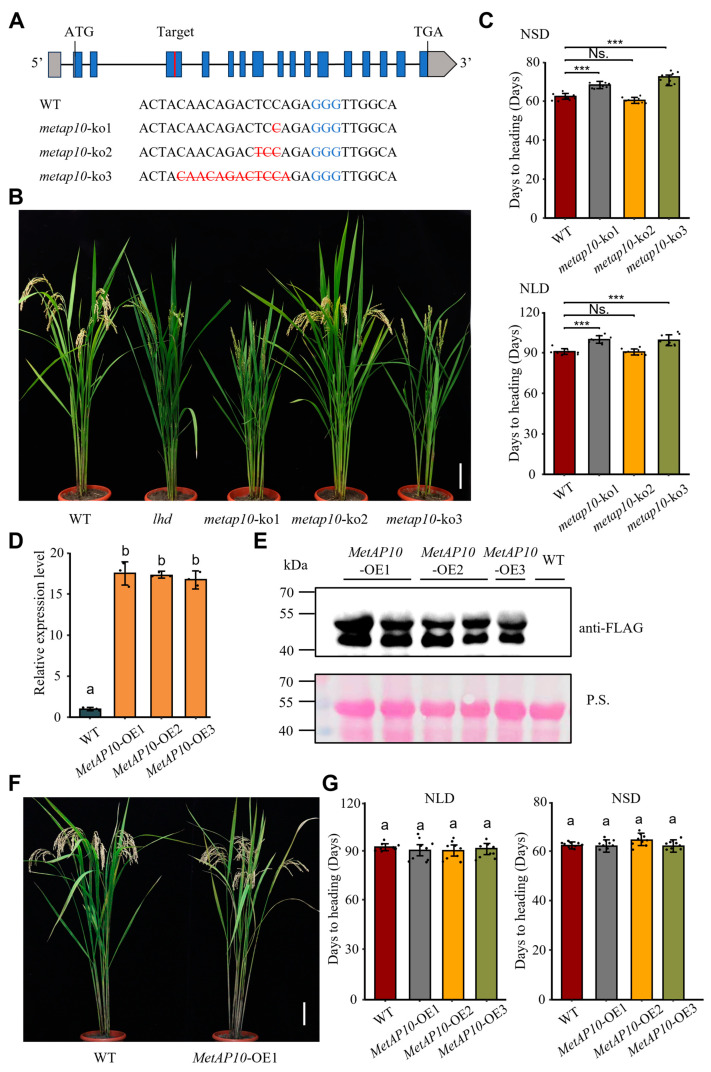
Functional verification of the *OsMetAP10* gene. (**A**) Identification of OsMetAP10 knockout lines generated by CRISPR/Cas9. Blue text indicates the target site of the guide RNA and the protospacer-adjacent motif (PAM). Red letters with a horizontal deletion line represent the deleted bases. (**B**) Plant phenotypes of wild-type (WT), *lhd* mutant, and *metap 10*-ko lines under the NLD condition. Scale bar = 10 cm. (**C**) Days to flowering of WT and *metap 10*-ko lines under NLD and NSD conditions. *** Indicates a statistically significant difference: *p* < 0.001 (Student’s *t*-test). Ns., no significant difference. (**D**) Relative expression levels of *OsMetAP10* in WT and MetAP10-OE plants. Values are presented as mean ± SD, and different letters indicate significant differences under Duncan’s multiple range test (*p* < 0.05). Data groups labeled with the same letter show no significant differences, while those labeled with different letters exhibit significant differences at <0.05 statistic level. (**E**) Expression of MetAP10-Flag protein in WT and MetAP10-OE plants. The upper panel shows the results of Western blot analysis, and the lower panel shows the Ponceau S staining results. (**F**) Plants of WT and MetAP10-OE1 under the NLD condition. Scale bar = 10 cm. (**G**) Days to flowering of WT and OsMetAP10 overexpression lines under NLD and NSD conditions. Small letter ‘a’ indicates no significant difference under Duncan’s multiple range test (*p* > 0.05).

To confirm if *OsMetAP10* associated with the late-heading phenotype of *lhd*, we employed CRISPR/Cas9 to knock out *OsMetAP10* in WT and obtained three homozygous knockout (ko) lines (Figure 4A and Figure S2A). Among them, the *metap10*-ko1 and *metap10*-ko3 mutants had deletions of 1 and 12 bases, respectively, both of which led to premature termination of protein translation. The *metap10*-ko2 had a deletion of three bases (TCC), resulting in a single proline deletion in the OsMetAP10 protein (Figure 4A and Figure S4A). Under both NLD and NSD conditions, we found that the *metap10*-ko1 and *metap10*-ko3 lines all exhibited late flowering compared to WT, which was consistent with the phenotype of the *lhd* mutant, while the *metap10*-ko2 showed no significant differences with the WT (Figure 4B,C). Compared with the WT, we also found that several agronomic traits, like plant height, panicle length, etc., of *metap10*-ko1 and *metap10*-ko3, but not of *metap10*-ko2, were significantly decreased, and showed a similar phenotype to the *lhd* mutant (Figure S4B and Table S2). These data demonstrated that the mutation of the *OsMetAP10* gene was responsible for the late flowering and bad agronomical performance of the *lhd* mutant.

Furthermore, we developed an overexpression construct for *OsMetAP10*, pUbi:OsMetAP10-Flag, and transformed it into WT (Figure S4C). Through RT-qPCR and Western blotting analysis, three homozygous lines with significant overexpression of the *OsMetAP10* gene were selected for further analysis (Figure 4D,E and Figure S5). We found that there was no significant difference in the heading dates between all three overexpression lines and WT under both NLD and NSD conditions (Figure 4F,G). However, these overexpression lines all showed significant reductions in plant height, grain number per panicle, and second branch number per panicle compared to the WT (Figure S4C and Table S2). These data indicated that *OsMetAP10* overexpression had no effect on the heading date but a significant effect on some agronomic traits. We concluded that *OsMetAP10* plays a critical role in maintaining normal flowering and the development of agronomically important traits under both LD and SD conditions.

### 3.5. Spatiotemporal Expression and Subcellular Localization of OsMetAP10 Protein

**Figure 5:**
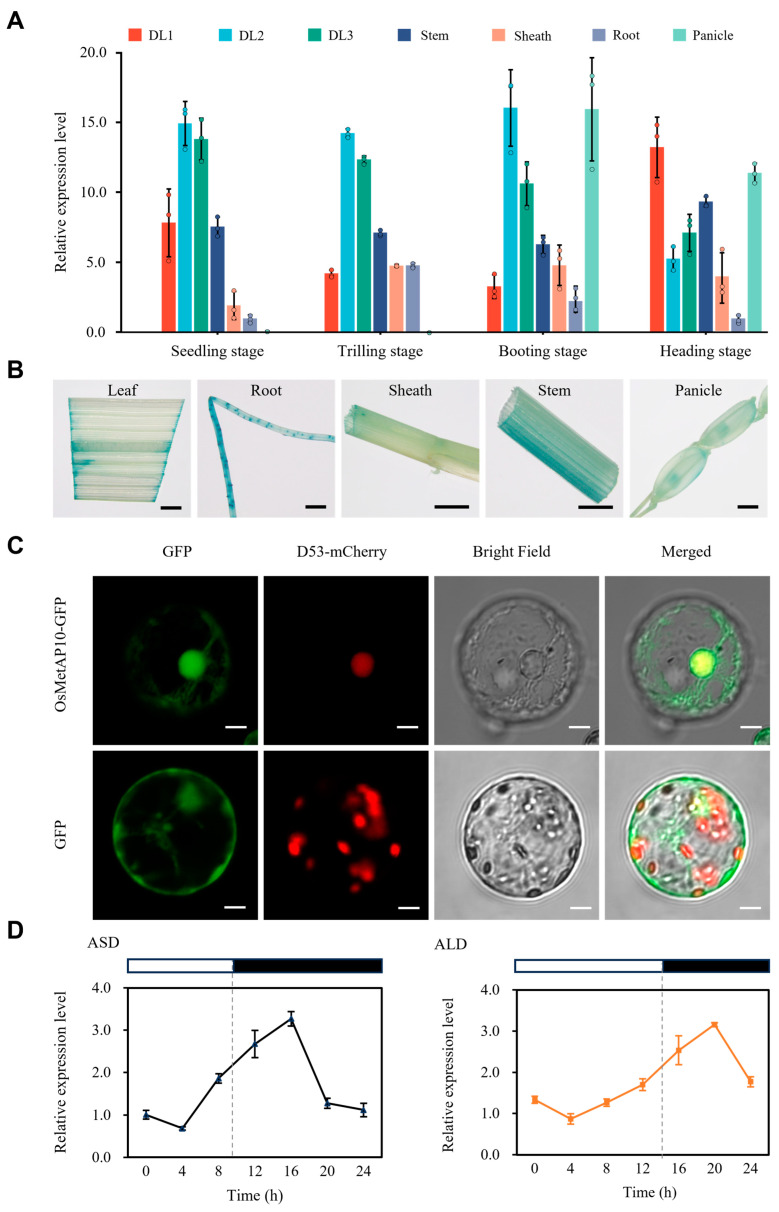
Expression patterns of OsMetAP10. (**A**) Relative transcription levels of *OsMetAP10* in different plant tissues at various development stages under ASD conditions. DL1, the first leaf from the top; DL2, the second leaf from the top; DL3, the third leaf from the top. (**B**) *GUS* expression (blue staining) patterns in different tissues of pOsMetAP10::GUS transgenic plants. Scale bar = 2 mm. (**C**) Subcellular localization of OsMetAP10 in rice protoplasts. As described in the previous literature, D53–mCherry was utilized as a nuclear marker [3,32]. GFP was used as the control. Scale bar = 5 μM. (**D**) Rhythmic expression pattern of *OsMetAP10* in WT under ASD and ALD conditions. The white rectangle indicates the light condition, and the black rectangle indicates the dark condition. The ubiquitin gene was used to normalize gene expression. Each time point represents the mean ± SD of three independent samples.

To explore the expression pattern of *OsMetAP10*, tissue samples from rice at different growth stages under the NLD condition were collected and analyzed by RT-qPCR. The results revealed that *OsMetAP10* was constitutively expressed in all different development stages in rice. The expression levels of *OsMetAP10* were highest in leaves, stems, and panicles, but relatively lower in roots and leaf sheaths (Figure 5A). Moreover, the tissue-specific expression of *OsMetAP10* was examined using the transgenic plants carrying a fusion protein between OsMetAP10 and GUS. Via the staining assay, *GUS* signals were detected in leaves, sheaths, stems, roots, and spikelets at different stages, further suggesting that *OsMetAP10* was constitutively expressed in different tissues (Figure 5B).

To identify the subcellular localization of OsMetAP10 protein function, an OsMetAP10-GFP fusion protein construct was generated and transiently expressed in the rice protoplast system. Confocal microscopy images clearly showed that OsMetAP10 was predominantly expressed in the nucleus and cytoplasm (Figure 5C).

Since the *OsMetAP10* gene was involved in the regulation of the heading date, we further tested if *OsMetAP10* presented a rhythmic expression. We collected leaf samples at four-hour intervals from WT plants under artificial long-day (ALD) and artificial short-day (ASD) conditions to monitor the expression change of *OsMetAP10* within a day. The results showed that under both SD and LD conditions, the expression of *OsMetAP10* began to increase at around 4 h after illumination and reached the expression peak at around 6 h after darkness, then gradually decreased (Figure 5D). Comparatively, the expression of *OsMetAP10* more sharply increased under the ASD condition than that in the SLD condition, while the peak levels were similar. Collectively, these data indicated that the expression of *OsMetAP10* was rhythmic.

### 3.6. Analysis of Downstream Signals of OsMetAP10 Mutation Leading to Late Heading

**Figure 6:**
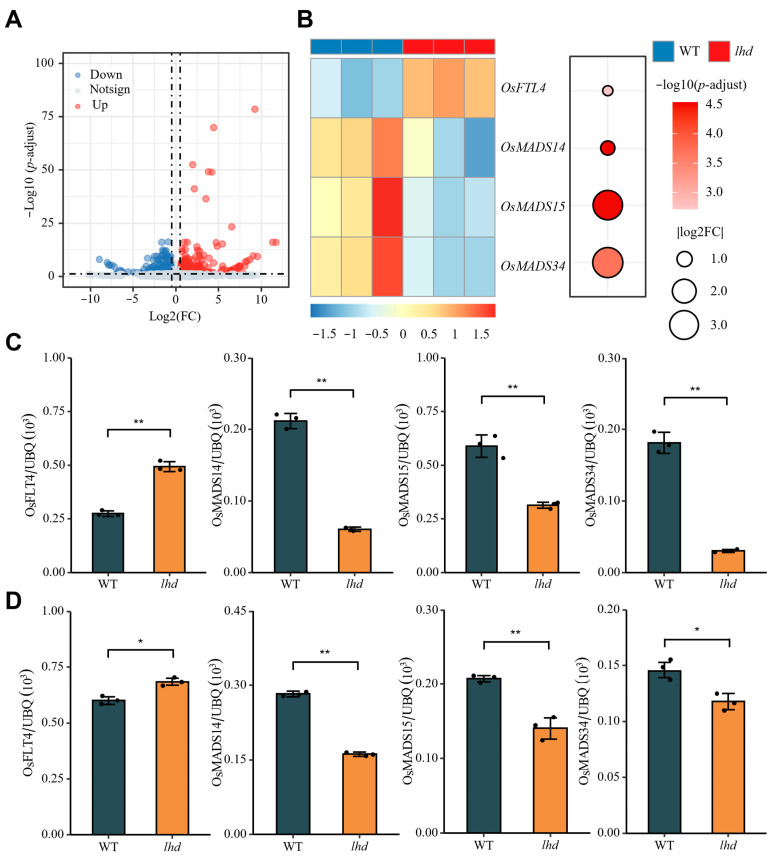
Expression analysis of heading date association genes in *lhd* mutant and wildtype plants. (**A**) Volcano plot of differentially expressed genes (threshold: Log_2_(fold change) > 0.5, *p*-adjust < 0.05) in the SAMs of WT and *lhd* under the ALD condition. (**B**) Expression patterns of *OsFTL4* and three flower development-related genes (*OsMADS14*, *OsMADS14*, and *OsMADS34*) in WT and *lhd*. The left panel shows the expression heatmap of the four genes. The color of bubbles in the right panel represents −log_10_(*p*-adjust), and the bubble size indicates the absolute value of Log_2_(FC). (**C**,**D**) Comparison of transcription levels of *OsFTL4*, *OsMADS14*, *OsMADS15*, and *OsMADS34* in WT and *lhd* under ALD (**C**) and ASD (**D**) conditions. The *ubiquitin* gene was used for gene expression normalization. * and ** indicate statistically significant differences with *p* < 0.05 and *p* < 0.01 (Student’s *t* test).

To explore the pathway by which the *OsMetAP10* mutation led to a delay in flowering, the SAMs of 4-week-old WT and *lhd* mutants under ALD conditions were collected for RNA-seq analysis. DEGs with Log_2_(fold change) > 0.5 and *p*-adjust < 0.05 were screened, leading to the obtainment of 963 DEGs (516 upregulated and 447 downregulated; Figure 6A). Four flowering-related genes were detected in DEGs, including a flowering repressor, *OsFTL4*, and three flower development-related genes, *OsMADS14*, *OsMADS15*, and *OsMADS34* (Figure 6B and Table S4). We then conducted RT-qPCR to verify their expression and obtained consistent trends to those in RNA-seq (Figure 6C). The *OsFTL4* gene was clearly upregulated in *lhd*, while the other three genes were all significantly downregulated compared with WT (Figure 6C). Because the *OsMetAP10* mutant led to a delay in heading regardless of NLD and NSD conditions, we further detected the expression of these four genes under the ASD condition and observed a consistent result to that in the ALD condition (Figure 6D). Together, the mutation of *OsMetAP10* may delay flowering by upregulating the expression of *OsFTL4* and downregulating the expression of *OsMADS14*, Os*MADS15*, and *OsMADS34*.

## 4. Discussion

The heading date is one of the critical agronomic traits in rice, which determines the planting region of a cultivar. To date, more than 100 QTLs associated with the heading date have been mapped, and many of them have been characterized. Nevertheless, due to the complexity of the heading date, the molecular mechanism underlying the trait remains to be elucidated, which requires identifying more genes involved in the regulation of flowering and dissecting their mechanisms. In this study, we identified a late-heading mutant, *lhd*, and confirmed that the mutation of the *OsMetAP10* gene accounted for its late-heading phenotype via the MutMap+ method and transgenic validation (Figure 2). *OsMetAP10* encodes a methionine aminopeptidase protein, but, due to the lack of systematic classification and identification for the protein in rice, the protein family remains unclear in rice development [22]. We then used the typical peptidase_M24 domain to search rice and *Arabidopsis* genomes and identified 15 rice genes and 12 *Arabidopsis* genes containing the domain. Through phylogenetic analysis, we inferred that OsMetAP10 belongs to peptidase_M24 protein subfamily III, in which all contain the motif 2 and motif 6 and are conserved in plant species (Figure 3C and Table S4). Although some studies have suggested the involvement of this gene family in plant growth, development, and abiotic stress responses, few genes have been well documented in rice [24]. Compared to WT, we found that *OsMetAP10* is required for the rice normal flowering time under both LD and SD conditions (Figure 1 and Figure 4). Also, we noted that several agronomic traits were severely affected by the gene mutation, indicating the essential role of *OsMetAP10* in rice development, which is consistent with the fact that *OsMetAP10* is constitutively expressed in various tissues and development stages (Figure 5). However, it is very interesting that *OsMetAP10* overexpression did not change the heading date (Figure 4). The closest Arabidopsis homologue of OsMetAP10, MAP1A, participates in the essential cytoplasmic NME process [23]. The *map1a*-ko plants and dsRNA interference, targeting *MAP1A* alone or both MAP2s (other types of minimal cytoplasmic processes), all exhibited wild-type phenotypes. However, under the situation of MAP2 inactivity, the growth and development processes of the *map1a*-ko plans were markedly suppressed, suggesting a trace level of MetAP protein is required for the cytoplasmic NME process [23]. In addition, *OsMetAP10* was constitutively expressed across developmental stages and in various tissues (Figure 5A,B), and this could be the reason that overexpression of the gene did not change the heading date nor the morphological phenotypes. Certainly, whether or not the *OsMetAP10* overexpression affects other undetected traits requires further investigation. Moreover, metap10-ko2 with only a proline deletion exhibited no changes in heading and other agronomic traits, except for a shorter plant height, suggesting the potential of *OsMetAP10* in finely modulating agronomic traits via gene editing in the future.

Previous studies have shown that during floral formation, the Hd3a and RFT1 proteins can be transported via the vascular bundles in the phloem to the SAM to induce flowering [5,7,10]. In the SAM cytoplasm, Hd3a and RFT1 proteins interact with 14-3-3, and then the complex moves to the nucleus to form a FAC with OsFD1, promoting *OsMADS14*, *OsMADS15*, *OsMADS18*, and *OsMADS34* transcription for floral transition [5,7,10]. To explore the reason for late heading from the *OsMetAP10* mutation, we conducted an RNA-seq analysis using the SAM tissue from WT and *lhd* plants under the ALD condition. We found that *OsFLT4* was upregulated, and *OsMADS14*, *OsMADS15*, and *OsMADS34* showed reduced expression in *lhd* plants (Figure 6). *OsFLT4*, like the repressor *RCN* gene, competes with Hd3a for 14-3-3 binding, inhibiting *OsMADS14*, *OsMADS15*, *OsMADS18*, and *OsMADS34* transcription [33,34]. The expression of *OsMetAP10* exhibited a circadian rhythm and was induced by light, suggesting its involvement in the regulation of the rice heading date (Figure 5C). The OsMetAP10 protein is located in both the cytoplasm and nucleus (Figure 5D), which is similar to the subcellular location prediction of TaM24 protein, as previously reported [22]. Therefore, we inferred that the later heading of *lhd*/*OsMetAP10* was due to the upregulation of *OsFLT4*. In the *OsMetAP10* mutant plants, more *OsFLT4* may compete with *Hd3a* for 14-3-3 interaction in the SAM, which further inhibits *OsFD1* binding with 14-3-3 and Hd3a to form a FAC, and then blocks the expression of *OsMADS14*, *OsMADS15*, and *OsMADS34*, finally resulting in a later flowering phenotype of *OsMetAP10/lhd* mutant than WT plants. However, how *OsMetAP10* affects the upregulation of OsFLT4 remains unclear, which is valuable to be further elucidated. Additionally, besides the contribution of *OsFLT4* upregulation to the later heading date, whether any other genes are involved in regulating the later flowering of *OsMetAP10* mutant plants is worth further study. Our study revealed a new role of *OsMetAP10* in regulating rice flowering, while the underlying regulation mechanism remains to be further investigated.

## 5. Conclusions

In this study, a peptidase_M24 superfamily gene, *OsMetAP10*, was confirmed to account for the late flowering of a rice *lhd* mutant. The OsMetAP10 protein was located in both cytoplasm and nucleus and was constitutively expressed across developmental stages and various tissues. The *OsMetAP10* mutation upregulated the expression of the negative flowering regulator *OsFLT4* and downregulated the expression of three key floral transition genes, *OsMADS14*, *OsMADS15*, and *OsMADS34*. Our study provided new insight into the pathways regulating rice heading.

## Data Availability

All data used to support the findings of this study are included within the article and are also available from the corresponding author upon request.

## Supplementary Materials

The following supporting information can be downloaded at: https://www.mdpi.com/article/10.3390/biology14020178/s1. Figure S1: Schematic diagram of the construction of the pCubi1390 vector. Figure S2: Distribution map of the ΔSnp index on 12 chromosomes. Figure S3: Protein sequences used for constructing the phylogenetic tree of the peptidase_M24 family in rice and *Arabidopsis thaliana*. Figure S4: Functional verification of OsMetAP10. Figure S5: Expression of MetAP10-3×Flag protein (52.58 kDa) in WT and MetAP10-OE plants. Table S1: The primer sequence information involved in this article. Table S2: Comparison of the main agronomic traits among the *lhd* mutant, OsMetAP10 knockout lines, overexpression lines, and the WT plant Dongjin. Table S3: Mutations identified by MutMap+ with the ΔInDel index. Table S4: Information on the motif sequence. Table S5: RNA-seq results of *OsFTL4* and three flower development-related genes (*OsMADS14*, *OsMADS14*, and *OsMADS34*) in WT and *lhd*.

## Abbreviations

The following abbreviations are used in this manuscript:

ND	Natural day
LD	Long day
SD	Short day
FT	FLOWERING LOCUS T
BVP	Basic vegetative growth phase
Hd3a	Heading date 3a
RFT1	RICE FLOWERING LOCUS T1
SAM	Shoot apical meristem
FAC	Flowering activation complex
OsGI	OsGIGANTEA
Hd1	Heading date 1
GI	GIGANTEA
CO	CONSTANS
DTH8	Days to heading on chromosome 8
Ghd7	Grain number, plant height, and heading date 7
Ehd1	Early heading date 1
OsCOL4	OsCONSTANS-LIKE 4
MAP	Methionine aminopeptidase
NME	N-terminal methionine (Met) excision
CLM38	Calmodulin-like protein 38
GUS	β-glucuronidase
lhd	Late-heading-date mutant
WT	Wild-type
NLD	Natural long day
NSD	Natural short day
ALD	Artificial long day
ASD	Artificial short day
HMM	Hidden Markov model
