# Supplementary material for: Isolation of OsMetAP10, a Peptidase_M24 Superfamily Gene, Regulating Heading Date in Rice
Source: Biology (Basel). 2025 Feb 10;14(2):178. doi: 10.3390/biology14020178 (PMC11851548; doi:10.3390/biology14020178)
Supplement: Supplementary file 1 [file biology-14-00178-s001.zip › biology-3436694-supplementary.pdf]

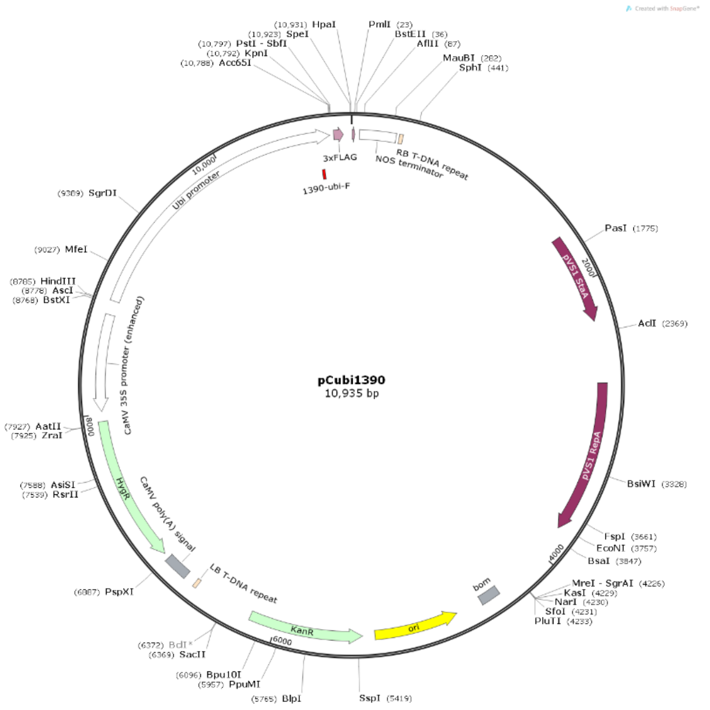

Figure S1. Schematic diagram of the construction of the pCubi1390 vector. 1390-ubi-F represents a primer used for sequencing.

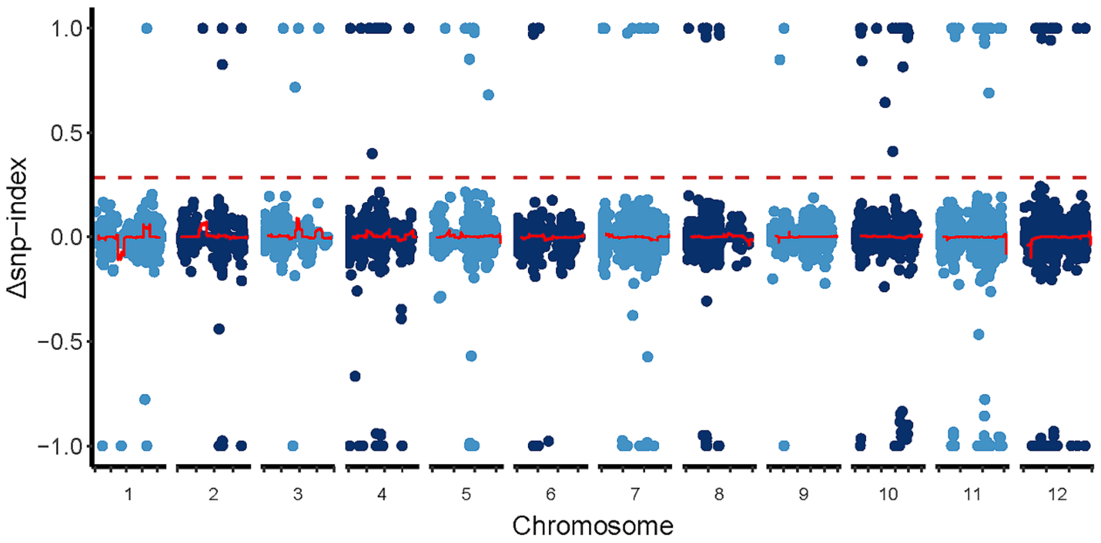

Figure S2. Distribution map of ΔSnp-index on 12 chromosomes. Points in the graphs represent SNPs, and the red lines represent the sliding window average of 5 Mb interval with 10 Kb increment. The threshold is represented by a reddish-brown dotted line and is obtained by calculating the average value of all ΔSnp-index plus three times the standard deviation of all ΔSnp-index(0.28).

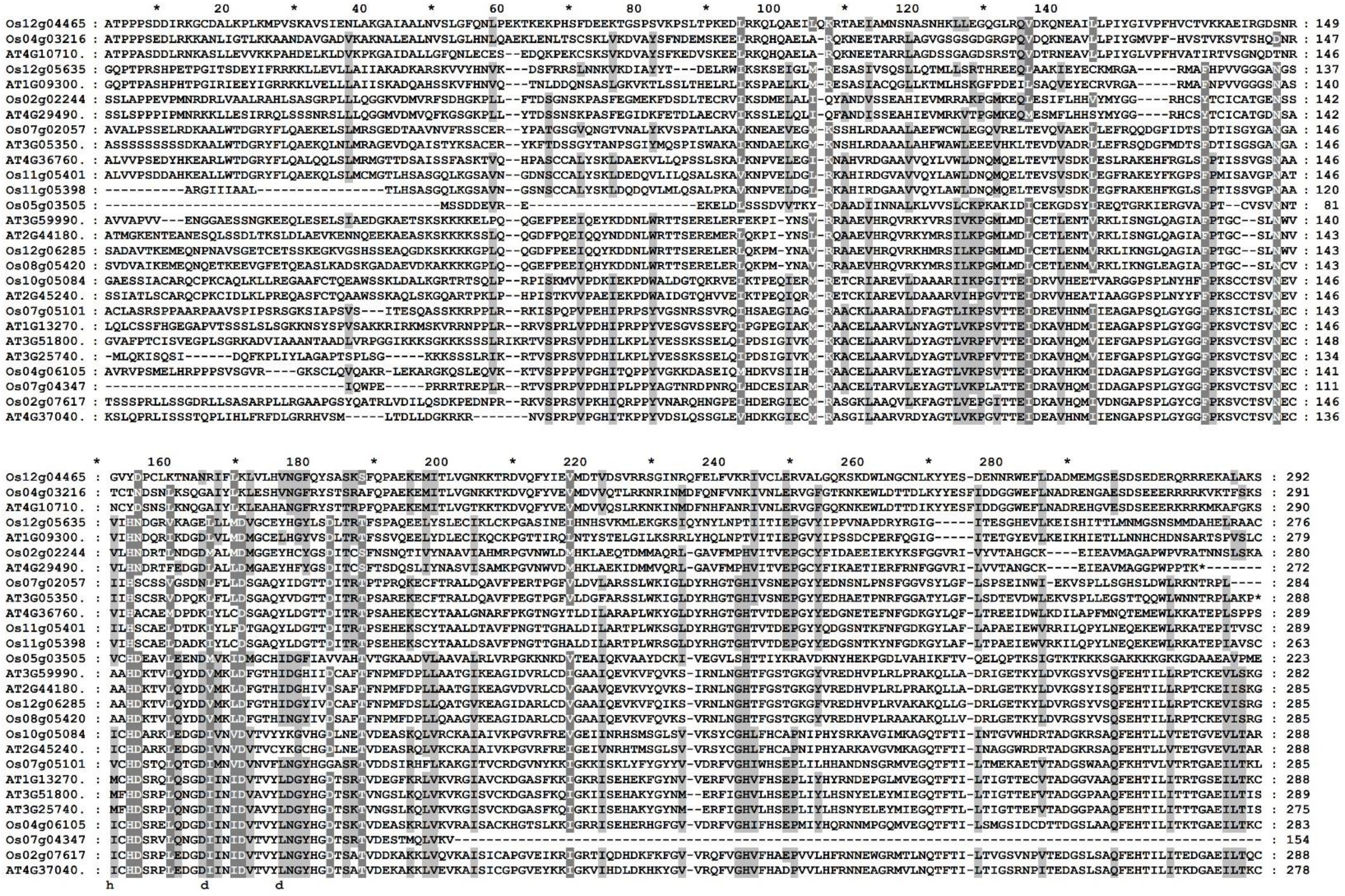

Figure S3. Protein sequences used for constructing the phylogenetic tree of the Peptidase_M24 family in rice and *Arabidopsis thaliana.*

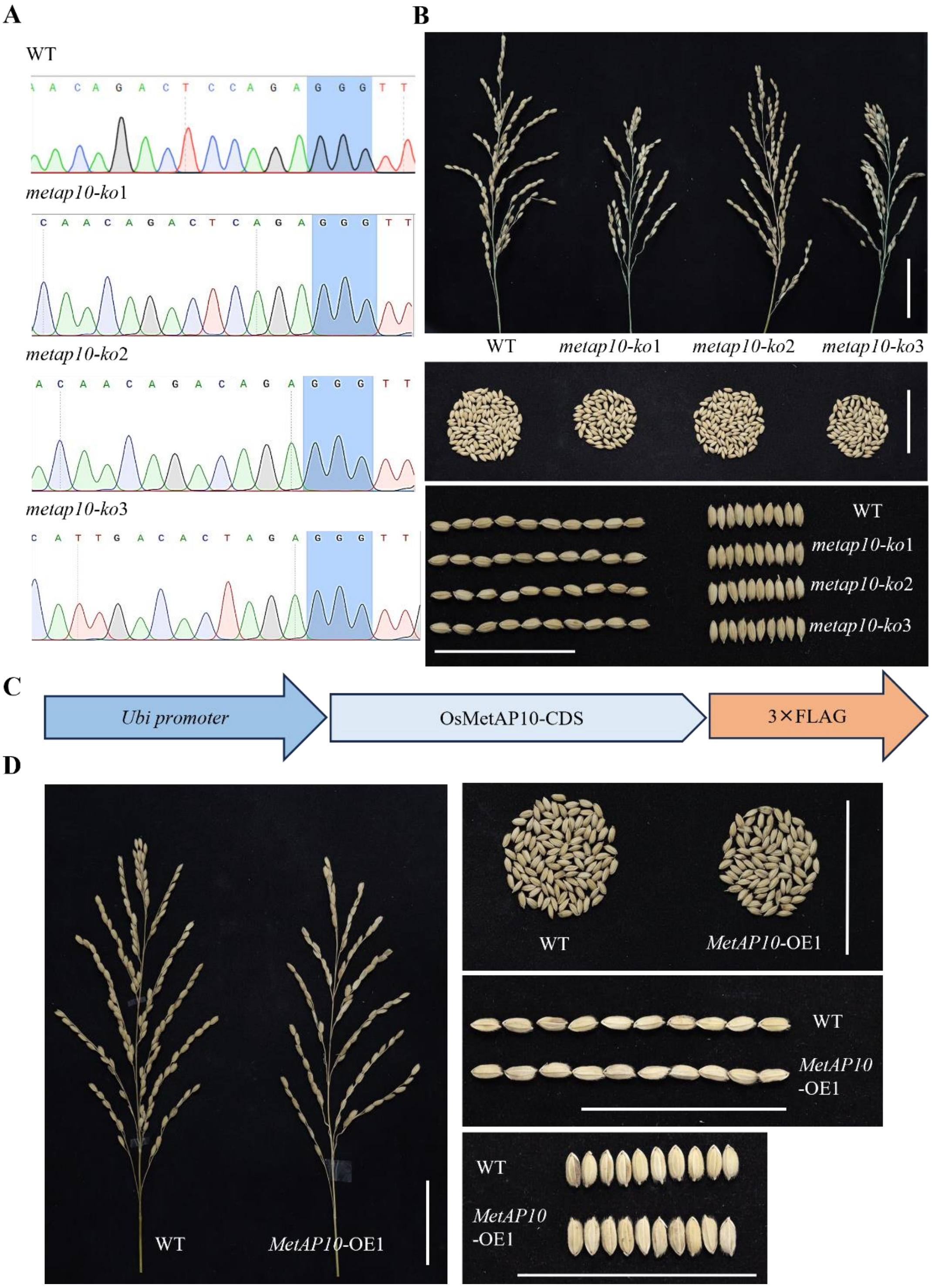

Figure S4. Functional verification of *OsMetAP10*. (A) Sequencing chromatograms near the target sites of the three knockout lines of *OsMetAP10*. (B) Photographs of panicles and grains of WT and the three metap10-ko knockout lines at the maturity stage. Scale bar = 5 cm. (C) Schematic diagram of the construction of the *OsMetAP10* overexpression vector. (D) Photographs of panicles and grains of WT and MetAP10-OE1 at the maturity stage.

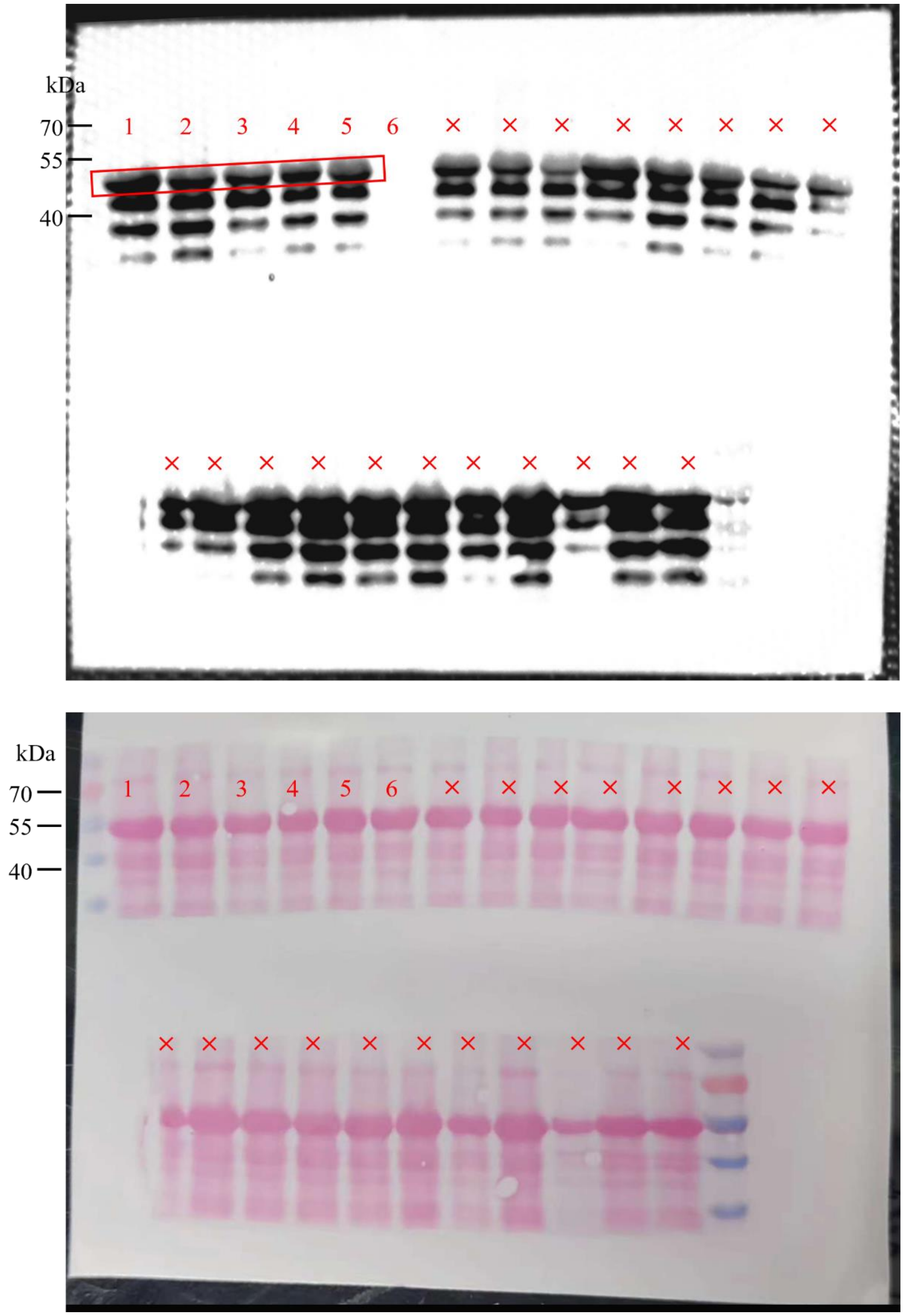

Figure S5. Expression of MetAP10-3 Flag protein(52.58kDa) in WT and MetAP10-OE plants. The upper panel shows the results of Western blot analysis, and the lower panel shows the Ponceau S staining results. The molecular weight of the protein is 52.58. The target protein band is within the red box, and the others are non-specific bands. Lane 1 - 2: MetAP10 Overexpression Line 1; Lane 3 - 4: MetAP10 Overexpression Line 2; Lane 5: MetAP10 Overexpression Line 3; Lane 6: Wild Type; Lane: Other materials.

Table S1 The primer sequence information involved in this article.

Primer name	Primer sequence(5'-3')	Note
8400-F	ACAGCACACAGGATTGCTTC	For detecting the variant sites of the OsMetAP10 gene.
8400-R	ACGAGCAAGCAAGTTTAGTAAGC	
1390ubi-F	CCCTGCCTTCATACGCTATT	Detect the positive plants of MetAP10-OE together with 8400-R.
1390-8400-F	cactaggtacctgcaATGGAGAAGGGGGGCGCC	Used for constructing the overexpression vector of OsMetAP10
1390-8400-R	gatccgtacctgcaTCACGGCTTCAACCATGGAAAGACATC	
p8400-GUS-F	tggctgcaggtcgacggatccATCCTATTGTAAATATCATG	Used for constructing the *GUS* expression vector of the OsMetAP10 promoter.
p8400-GUS-R	ggactgaccacccggggatccAAGCTGCTCTGCTCCCCAC	
qEhd1-F	AACCCGGTCATCCTCCAT	For RT-qPCR of Heading-related genes
qEhd1-R	TCATCTCTCACCTCATTTTCT	
qHd1-F	CGTTTCGCCAAGAGATCAG	
qHd1-R	AGATAGAGCTGCAGTGGAGAAC	
qRFT1-F	GTCGCCACCGTCTACTTCAA	
qRFT1-R	ATACAGCTAGGCAGGTCTCAG	
qHd3a-F	GTCTACCCCTAGCTAACGATGA	
qHd3a-R	CACCATCATATATATGTTGTGTGTCG	
qDTH2-F	CCAGTTTCAACGACGCCTAA	
qDTH2-R	GTCTCCATATACGCTCCCATCA	
qPHYB-F	CTCATCTTCAAGGAATCTGAGG	
qPHYB-R	CCTGCTAGAACAAGCATTCAC	
qOsGI-F	ATCGTTCTGCAGGCCGAGA	
qOsGI-R	TCACCAATGCTTCTGGGCTAT	
qGhd7-F	AATCCGGTACGCGTCCAGA	
qGhd7-R	CCAAGCTCAAGCCTACTAGG	
qPHYC-F	AAGGGCCTATTGTGCTCCAAGTC	
qPHYC-R	AGCTGGATGGACAAGCCTGAAC	
qCOL4-F	GTCCATGGACGGAATCAAGG	
qCOL4-R	CTCCGACGACGACAAGCTGT	
qSE5-F	AGGACTCCCAAGCTTTTATC	
qSE5-R	CTCCAGAATACGAGAACGAC	
qPRR37-F	CGGCAGTGGCAATGACATGT	
qPRR37-R	CCTCTTTCTGCTCTGGTACC	

Table S2 Comparison of the main agronomic Traits among the *lhd* mutant, OsMetAP10 knockout lines, overexpression lines and the WT plant Dongjin

Material	Dongjin	*lhd*	*metap10*-ko1	*metap10*-ko2	*metap10*-ko3	MetAP10-OE1	MetAP10-OE2	MetAP10-OE3
Flag leaf length（cm）	41.61±3.1	40.44±4.4	40.11±4.88	40.89±2.75	40.94±2.16	42.09±3.46	40.4±3.11	41.97±3.75
Flag leaf width(cm)	1.64±0.06	1.66±0.05	1.64±0.07	1.72±0.12	1.65±0.05	1.68±0.07	1.66±0.06	1.67±0.08
Grain number per panicle	136.44±15.22	83.33±13.63**	78.44±27.42**	134.22±23	91.89±14.7**	100.67±19.07**	106.89±17.51**	106.33±16.62**
Seed setting rates(%)	0.94±0.02	0.84±0.02**	0.68±0.12**	0.92±0.03	0.6±0.21**	0.95±0.02	0.96±0.02	0.95±0.02
Panicle length(cm)	21.28±1.45	19.42±2.28	17.56±2.21**	20.39±1.9	18.07±1.26**	20.08±2.19	19.87±1.13	20.81±1.72
Primary branch number per panicle	11.56±1.42	11.11±1.69	10.11±1.27*	11.11±1.17	10.22±1.72*	11.22±1.39	11.22±1.39	11±1.58**
Second branch number per panicle	24.73±2.27	18.44±4.19**	6.89±2.67**	21.56±4.72	4.89±1.9**	13.33±2.6**	15.11±1.45**	14.89±2.09**
Plant length(cm)	106±2.88	97.32±2.9**	83.44±3.74**	89.5±3.16**	67.88±4.1**	97±3.07**	99.13±3.58**	98.63±4.38**
Grain length(cm)	7.52±0.43	7.59±0.36	7.59±0.26	7.43±0.35	7.52±0.31*	7.33±0.31	7.72±0.28	7.73±0.33
Grain width(cm)	3.65±0.2	3.59±0.2	3.55±0.14	3.64±0.19	3.61±0.2	3.55±0.21	3.75±0.18	3.74±0.14

Note: Data are presented as mean ± standard error. The LSD test was used for significance analysis. * and ** represent significant differences at the 0.05 and 0.01 levels, respectively. Dongjin was used as the control.

Table S3 Mutations identified by MutMap$^+$ with Δ InDel index.

Chromosome	Position	Reference	Altered base	Type of mutation	Involved gene
10	19451801	G	GCCT	Nonframeshift insertion	*Os10g0508000* (Monocopper oxidase)
10	19513884	TC	T	Stopgain	*Os10g0508400* (Peptidase M24)
10	19817733	G	GT	Intergenic	Not applicable
10	20022960	C	CTTG	Intergenic	Not applicable

Note: Nonframeshift insertion indicates that the number of inserted bases is a multiple of three, which will not cause a misreading of subsequent codons

Table S4 Information of Motif Sequence

Motif	Sequence
motif 1	VSLNAAVAHYSPEAGDCTVLDYDDVMKLDSGAHIDGGITDIARTVHFNEP
motif 2	WTAVTADGSYAAQFEHTILITPTGAEILT
motif 3	TNTGIKEAGIDVRLCDVGAAIQEVMESYEVEINGKVFQVKSIRNLNGHSI
motif 4	CYTAVLKGHIALDQAVFPEGTPGFVLDILARSPLWKIGLDYRHGTGHGVG
motif 5	LVKVTEECLEKAISICKPGVSFKKIGKIISEHAAKYGYGVVR
motif 6	KSELZIHDKEGINRMRAACELAARVLDYAGTJVKPGVTTDEIDEAVHNMI
motif 7	YQIHAGKSVPIVKGGEQTKMEEGEFYAIETFGSTGKGYVREDLECSHYMK
motif 8	KKKKSKSKKKKGPLQQTDPPSIPVDELFPSGDFPEGEIQQYKDDNLWRTT
motif 9	FDVGHVPLRLPKAKQLLATINKNFGTLAFCRRYLDRLGETKYLMALKNLC
motif 10	YPSPLGYGGFPKSVCTSVNECICHGIPDSRPLZDGDIINID

Table S5 RNA-seq results of OsFTL4 and three flower development-related genes (*OsMADS14*, *OsMADS14* and *OsMADS34*) in WT and *lhd*.

Gene name	WT-1	WT-2	WT-3	*lhd*-1	*lhd*-2	*lhd*-3	Log_2FoldChange	p-adiust
OsFTL4	6.11	6.05	6.07	6.26	6.28	6.25	0.84	0.00193
OsMADS14	12.26	12.35	12.61	12	11.64	11.41	-0.93	0.0000289
OsMADS15	5.66	5.77	6.22	5.48	5.34	5.41	-3.03	0.0000314
OsMADS34	2.74	2.77	2.84	2.67	2.64	2.64	-3.17	0.0002

Note: Each group has three biological replicates. The heatmap is drawn using TPM data. The differential gene expression analysis is calculated using count values.
